# Biomarkers of Animal Nutrition: From Seasonal to Lifetime Indicators of Environmental Conditions

**DOI:** 10.3390/life12030375

**Published:** 2022-03-04

**Authors:** Rachel A. Smiley, Tayler N. LaSharr, Heather N. Abernathy, Yasaman N. Shakeri, Rebecca L. Levine, Seth T. Rankins, Rhiannon P. Jakopak, Rebekah T. Rafferty, Jaron T. Kolek, Brittany L. Wagler, Samantha P. H. Dwinnell, Timothy J. Robinson, Jill E. Randall, Rusty C. Kaiser, Mark Thonhoff, Brandon Scurlock, Troy Fieseler, Gary L. Fralick, Kevin L. Monteith

**Affiliations:** 1 Haub School of the Environment and Natural Resources, University of Wyoming, 804 E Fremont St., Laramie, WY 82072, USA; tlasharr@uwyo.edu (T.N.L.); habernat@uwyo.edu (H.N.A.); yshakeri@uwyo.edu (Y.N.S.); rlevine1@uwyo.edu (R.L.L.); srankins@uwyo.edu (S.T.R.); rjakopak@uwyo.edu (R.P.J.); rraffer1@uwyo.edu (R.T.R.); jkolek@uwyo.edu (J.T.K.); bwagler@uwyo.edu (B.L.W.); kevin.monteith@uwyo.edu (K.L.M.); 2 Wyoming Cooperative Fish and Wildlife Research Unit, Department of Zoology and Physiology, University of Wyoming, 1000 E University Ave., Laramie, WY 82071, USA; 3 Arctic Terrestrial Biology, The University Centre in Svalbard, P.O. Box 156 N−9187, 9170 Longyearbyen, Norway; samanthad@unis.no; 4 Department of Mathematics & Statistics, University of Wyoming, Department 3036, 1000 E University Ave., Laramie, WY 82071, USA; tjrobin@uwyo.edu; 5 Wyoming Game and Fish Department, Pinedale Regional Office, 432 Mill St., Pinedale, WY 82941, USA; jill.randall@wyo.gov (J.E.R.); brandon.scurlock@wyo.gov (B.S.); troy.fieseler@wyo.gov (T.F.); 6 United States Forest Service, Big Piney Ranger District, 10418 South US Highway 189, Big Piney, WY 83113, USA; rusty.kaiser@usda.gov; 7 Bureau of Land Management, Pinedale Field Office, 1625 West Pine St., Pinedale, WY 82941, USA; mthonhof@blm.gov; 8 Wyoming Game and Fish Department, Jackson Regional Office, 420 North Cache, Jackson, WY 83001, USA; gary.fralick@wyo.gov

**Keywords:** biomarker, body fat, body mass, ingesta-free body fat, intraclass correlation coefficient, lean mass, nutrition, nutritional biomarker

## Abstract

Nutrition underpins survival and reproduction in animal populations; reliable nutritional biomarkers are therefore requisites to understanding environmental drivers of population dynamics. Biomarkers vary in scope of inference and sensitivity, making it important to know what and when to measure to properly quantify biological responses. We evaluated the repeatability of three nutritional biomarkers in a large, iteroparous mammal to evaluate the level of intrinsic and extrinsic contributions to those traits. During a long-term, individual-based study in a highly variable environment, we measured body fat, body mass, and lean mass of mule deer (*Odocoileus hemionus*) each autumn and spring. Lean mass was the most repeatable biomarker (0.72 autumn; 0.61 spring), followed by body mass (0.64 autumn; 0.53 spring), and then body fat (0.22 autumn; 0.01 spring). High repeatability in body and lean mass likely reflects primary structural composition, which is conserved across seasons. Low repeatability of body fat supports that it is the primary labile source of energy that is largely a product of environmental contributions of the previous season. Based on the disparate levels in repeatability among nutritional biomarkers, we contend that body and lean mass are better indicators of nutritional legacies (e.g., maternal effects), whereas body fat is a direct and sensitive reflection of recent nutritional gains and losses.

## 1. Introduction

Variations in physiological, morphological, and behavioral traits constitute the raw material for evolutionary change to act upon [1,2,3,4]. Though variation among individuals is a ubiquitous characteristic across taxa [5,6], sources of individual variation can be intrinsic (e.g., genotypic) or extrinsic (e.g., cultural, environmental) [7]. Understanding sources of individual variation may improve predictions regarding species responses to ecological and environmental drivers [5,8].

Repeatability in traits across time may serve to elucidate drivers of individual variation. Repeatability—the proportion of phenotypic variance explained by differences between individuals [9]—can be used to identify genetic, behavioral, or cultural heritability. Repeatability works to distinguish between intrinsic and extrinsic sources of individual differences [10], whereby repeatable traits are consistent within individuals across time, despite population and environmental variation; therefore, such traits are likely heritable [11]. Alternatively, if individual traits are inconsistent across time (i.e., low repeatability), then trait variation is likely influenced by extrinsic factors (e.g., environment) [12]. The challenges of isolating physiological processes associated with a trait or identifying and quantifying meaningful environmental drivers have led to the use of proxies in examining sources of individual variation (e.g., biomarkers) [9,13]. In this context, the propensity for biomarkers to be repeatable may provide context for identifying the responsiveness to intrinsic or extrinsic factors and the scope of inference that can be drawn from a biomarker.

Elsewhere, biomarkers have been used as proxies to make inferences regarding physiological states and nutritional conditions [14,15,16]. Biomarkers can reveal the underlying drivers of behavior, fitness, and responses to environmental change. For example, brown trout (*Salmo trutta*) in good nutritional condition, as measured by a ratio-based biomarker (mass/length^3^), migrated earlier than fish in poor condition [17]. Notably, however, biomarker selection may influence inferences regarding the physiological process under investigation. For instance, when examining biomarkers of hematocrit and lipid metabolites, neotropical birds showed reduced nutritional condition in response to drought; however, changes in condition were not detectable when scaled mass index was used as a biomarker [18]. Thus, the effective use of biomarkers hinges on both understanding drivers of variation within and across individuals and an understanding between the biomarker and the associated physiological process being measured.

Nutrition ultimately underpins fitness [19,20] and is especially important for species that partially depend on stored reserves to finance survival or reproduction [21,22]. Biomarkers of nutritional condition may help reveal the underlying drivers of individual and population-level fitness [23]. Nevertheless, subtle differences among biomarkers of nutrition may have a drastic influence on the inference that can be drawn. Body mass, a nutritional biomarker that is commonly used, integrates accumulation and catabolism of both body fat and lean mass (i.e., protein reserves), with protein reserves generally conserved over fat reserves [24]. Body fat is considered a labile source of energy and a product of the environment an animal experiences [24]. As a primary energetic store, body fat is regulated in a risk-sensitive manner; environmental conditions and nutritional state determine the degree to which individuals allocate fat reserves to survival and reproduction [25,26,27]. Indeed, for animals in temperate environments, body fat fluctuates seasonally with resource availability [24]. High-quality and abundant forage allows animals to accumulate high levels of fat during the summer, which can deplete when animals are faced with resource scarcity and harsh winter conditions [28]. Alternatively, lean mass represents the fat-free, ingesta-free mass of an animal and, thus, primarily reflects protein reserves. Protein is often conserved over fat, in part because protein also serves a structural value to the animal [27]. Consequently, fluctuations in protein may be subtle but are most obvious during the winter when animals are faced with both reductions in available forage and the physiological requirements of gestation. Given the physiological differences in how protein and fat are used during different seasons, estimates of body fat, lean mass, and body mass may reflect different life-history processes at different scales. Further, interpretation of these three biomarkers may differ in intrinsic (e.g., genotype) or extrinsic (e.g., environmental) expression. 

Biomarkers of nutrition likely are influenced by different biological processes and serve slightly different physiological roles. Therefore, differences in sensitivity to environmental conditions, and, thus, the scope of inference, likely exist between body fat, lean mass, and body mass. Nevertheless, various nutritional biomarkers are often used interchangeably as indicators of nutritional status [29,30]. We suspect that the repeatability of each biomarker could yield insight into the context or scope of inference that each biomarker could represent, which could improve their interpretation in ecological studies. Since traits with high repeatability are more heritable, individual variation within repeatable traits providing fitness benefits is likely to proliferate into the population. For example, if lean mass and body mass are more heritable, favorable individual variation in these traits (e.g., the correlation between body size and reproductive success) [31] would naturally be incorporated into the population through natural selection. Yet, highly heritable or canalized traits are often static among individuals and may reduce fitness by way of an inability for individuals to respond to stochasticity, therefore being maladaptive in certain contexts [32]. Given that some aspects of nutrition can act as a buffer against environmental stochasticity [25,33], the degree of heritability versus plasticity in nutritional biomarkers likely has consequences for their relevance and interpretation relative to environmental variation.

Using a long-term dataset of a large mammal through time, we sought to estimate the repeatability of three nutritional biomarkers (i.e., body fat, lean mass, and body mass) during periods of resource abundance and scarcity. Mule deer (*Odocoileus hemionus*) are highly fecund, long-lived mammals that are finely tuned to the heterogeneous landscapes that they occupy [34]. In the summer, when resources are abundant, mule deer must balance energy allocation between raising offspring and accruing somatic resources that were depleted the previous winter. Mule deer rely on fat and protein stores during the resource-limited winter months to finance their own survival and future reproduction [22]. Consequently, measures of nutritional condition are typified by seasonal fluctuations that often peak in autumn and drop in late winter and early spring. Nutritional biomarkers and their variation across seasons and years could affect reproduction or survival in a given year, as well as carry over to affect reproduction or survival in the future through maternal effects [35,36,37,38]. 

We hypothesized that the physiological processes that affect accretion and catabolism of somatic reserves influence the degree of repeatability of nutritional biomarkers (H1). Because lean mass reflects protein reserves and skeletal structure of an animal and is the last reserve to be catabolized, we predicted that it would be the most repeatable, followed by body mass and then body fat. We expected body fat to be the least repeatable because it is the most readily catabolized somatic store (P1). Next, given that energetic demands associated with the physiological processes vary between autumn and spring, we hypothesized that repeatability would vary between seasons (H2). We predicted that body fat would be more repeatable in spring than in autumn because of the potential variability of energetic investments to reproduction in autumn (P2). When mule deer replenish somatic reserves during summer, they prioritize protein stores before body fat and reproduction; therefore, we predicted both lean mass and body mass would be more repeatable in autumn than spring (P3). 

## 2. Materials and Methods

### 2.1. Study Area

We studied a population of migratory mule deer located in western Wyoming, USA (42°25′ N, 110°42′ W), a semi-arid region of the Rocky Mountains characterized by cold winters and warm, productive summers. Animals migrated to winter ranges from October to November and spent winters at low elevations (~2000–2300 m) in sagebrush-steppe ecosystems dominated by sagebrush species (*Artemisia tridentata*, *Artemisia nova*) [39]. Mean annual snowfall on winter ranges (Western Regional Climate Center [40] full record averages) varied from 68.61 cm in the north (Fontenelle Dam, Wyoming, 1977 m) to 196.14 cm in the south (Fossil Butte, Wyoming, 2068 m) [40]. The timing of spring green-up, from March to May, triggered spring migration to higher elevation summer ranges (~2000–3040 m) in the Salt and Wyoming Ranges, where individuals occupied a variety of vegetative communities along a steep elevational gradient [41,42]. Mean annual precipitation on summer ranges (Applied Climate Information System [43] 20-year averages) was 99.84 cm (Triple Peak, SNOTEL site, 2591 m [43]). Summer range vegetation types included low-elevation (~2000–2300 m) sagebrush communities similar to those on the winter range, middle elevation (~2300–2750 m) aspen (*Populus tremuloides*), mixed conifer, mixed mountain shrub and forb communities, and high-elevation (>2750 m) subalpine tall forb communities and pine-fir complexes [42]. There is no supplemental feeding in the population. The peak of parturition occurred in mid-June in the summer range.

The study period (2013–2021) coincided with highly variable weather conditions in western Wyoming, with multiple years experiencing snowfall that was well above average and some years with periods of extreme drought. During 2013–2021, annual snowfall (measured December through April) ranged from 12.70 cm at Fontenelle Dam, Wyoming in the winter of 2014–2015 to 236.22 cm in Fossil Butte, Wyoming in the winter of 2016–2017, the third greatest annual winter snowfall recorded for the state. Annual precipitation during the primary growing season (measured June through August, Triple Peak, SNOTEL site, 2591 m) ranged from 7.62 cm in 2016 to 20.07 cm in 2015. 

### 2.2. Data Collection

We captured adult (>2 yrs old) female mule deer twice a year using a net gun deployed via helicopter [44] every autumn (December) and spring (March) from December 2013 to March 2021. Recapture rates for this population are generally high, and most animals were captured at each capture event between their initial capture and either the end of the study or their death. We fitted captured deer with GPS collars (Advanced Telemetry Systems, Isanti, MN; Vectronics Aerospace, Berlin, Germany). We assigned a unique animal identification number to each animal, and, at each capture, animals were assigned an animal-season identifier (e.g., the animal-season for animal “001” in autumn 2015 would be “001-Autumn-2015”). We used manual palpation and ultrasonography (5-MHz transducer; Ibex Pro, E.I. Medical Imaging, Loveland, CO, USA) to estimate the ingesta-free body fat (hereafter, body fat; %) with standardized equations developed for mule deer [45,46]. One observer performed all body fat measurements throughout the study, so there was minimal variation in observer error. We estimated the body mass of deer using a platform scale (±0.1 kg). We calculated ingesta-free, fat-free body mass (hereafter, lean mass) by calculating ingesta-free body mass [46] and subtracting the weight of body fat (ingesta-free body mass x ingesta-free body fat) [24].

### 2.3. Statistical Analyses

Repeatability (i.e., the intraclass correlation coefficient) is the proportion of variance that can be attributed to the variance between animals and, thus, is estimated using both within-animal and between-animal variance estimates (Equation (1)) [47,48]. Repeatability estimates range from 0 to 1.0, and are considered low from 0 to 0.3, moderate from 0.3 to 0.5, and high from 0.5 to 1.0 [49]. We let $\sigma_{w}^{2}$ denote within-animal variance and $\sigma_{B}^{2}$ denote between-animal variance.

(1) $$\mathrm{Repeatability}=\frac{\sigma_{B}^{2}}{\sigma_{B}^{2}+\sigma_{W}^{2}}$$

We calculated the repeatability of three nutritional biomarkers (i.e., body fat, body mass, and lean mass) for mule deer using the ratio between variance components derived from Bayesian hierarchical models. We implemented the same model for each biomarker and two different seasons (autumn and spring) for a total of six separate models. Given repeatability is calculated using both within- and between-animal variance components, we only included animals in the analyses if they were captured more than once for a given season (e.g., an animal had to be captured in at least two springs to be included in the spring model). We evaluated biomarkers in each season as a function of a random intercept for individuals with a fixed mean. 

We assumed $y_{ij}|\mu_{ij},\sigma_{W}^{2}\sim N\left(\mu_{ij},\sigma_{W}^{2}\right)$, where $y_{ij}$ denotes the nutritional biomarker at time *j* for animal *i*. We assumed that the conditional mean response (conditional on animal *i*) is given *β*.

$$\mu_{ij}|\delta_{i}=\beta_{0}+\delta_{0i}$$

where *β*_0_ denotes a fixed-effects intercept and $\delta_{0i}$ represents the random deviation from the fixed effects intercept associated with animal *i*. Random intercepts were assumed to be normal (0,$\sigma_{B}^{2}$). 

We implemented our models examining the repeatability of three nutritional biomarkers using the WinBUGS [50] software in program R [51] with the ‘R2WinBUGS’ package [52]. Using the WinBUGS defaults, there was a burn-in of 1000 draws. We generated 100,000 draws and took from every 10th draw. We used the delta and epsilon variance estimates from the 10,000 draws to calculate repeatability estimates in the WinBUGS model. Results for the remainder of the analyses associated with repeatability are based on the 10,000 draws of the parameters. Models were considered convergent if they had unimodal posteriors, Ȓ values < 1.1 [53], and an even mixture of the MCMC chains [54]. For the Bayesian estimates reported, we used the median of the posteriors since the posteriors for body fat in spring were skewed right. Model formulation and corresponding code used for our Bayesian analyses can be found in the Supplementary Materials.

Repeatability for body fat in spring was low, which was partially because of minimal between-group variation (see Results). Given the low population-level variance of spring fat in the model, we evaluated additional descriptive statistics for both autumn and spring body fat to investigate this biomarker as an indicator of nutrition in different seasons. We calculated the mean, standard deviation, and coefficient of variation of body fat for each independent season (e.g., spring 2014 or autumn 2020) and averaged those three statistics across all years for both spring and autumn.

## 3. Results

We evaluated 1039 capture events for 182 adult female mule deer between autumn 2013 and spring 2021. We evaluated repeatability in autumn using 456 animal-seasons from 146 animals measured between 2 and 8 times (mean = 3.12). We evaluated repeatability in spring using 583 animal-seasons from 174 animals measured between 2 and 9 times (mean = 3.35). The recapture rate for individuals was extremely high, with a mean of 1.01 ± 0.01 years between spring captures and 1.02 ± 0.01 years between autumn.

**Figure 1:**
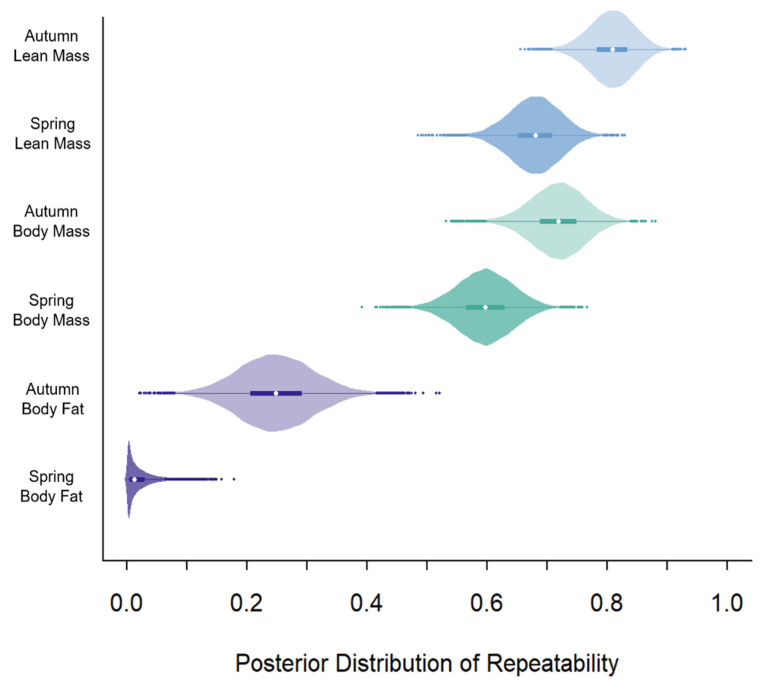
Posterior distribution of the repeatability estimates of lean mass (blue), body mass (green), and body fat (purple) in autumn and spring of female mule deer in the Wyoming and Salt Ranges from 2013 to 2021. Median is represented by the white dot, 50% credible intervals are represented by the thick colored bar, 95% credible intervals are represented with the thin, solid line, and outliers are represented by colored dots.

All models converged based on unimodal posteriors, Ȓ values < 1.1, and an even mixture of the MCMC chains. Across seasons, repeatability was greatest for lean mass, followed by body mass, and lowest for body fat (Table 1; Figure 1). The repeatability of all three biomarkers was greater in autumn than in spring (Table 1; Figure 1).

**Figure 2:**
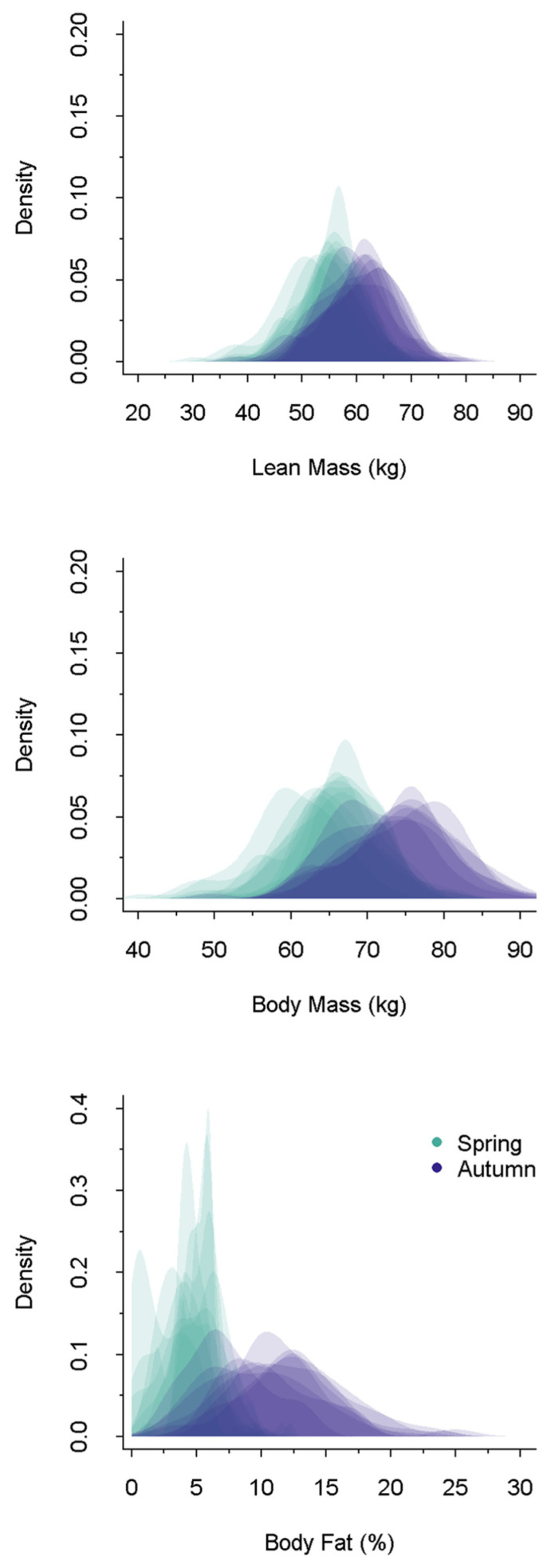
Annual density plots of lean mass (i.e., ingesta-free, fat free mass; kg), body mass (kg), and body fat (i.e., ingesta-free body fat; % IFBFat) in spring and autumn of mule deer in the Wyoming and Salt Ranges from autumn 2013 to spring 2021. Spring is represented by green density plots and autumn is represented by purple density plots. Please note, the *y*- and *x*-axes differ across all three biomarkers.

Within-animal variance was greatest for body fat, followed by body mass, and then lean mass across seasons (Table 2). Body fat was higher in autumn (mean = 10.82, Standard Deviation [SD] = 3.86) than spring (mean = 4.49, SD = 1.84), and the coefficient of variation was higher in spring (CV = 44.59) than in autumn (CV = 36.27; Figure 2).

## 4. Discussion

**Figure 3:**
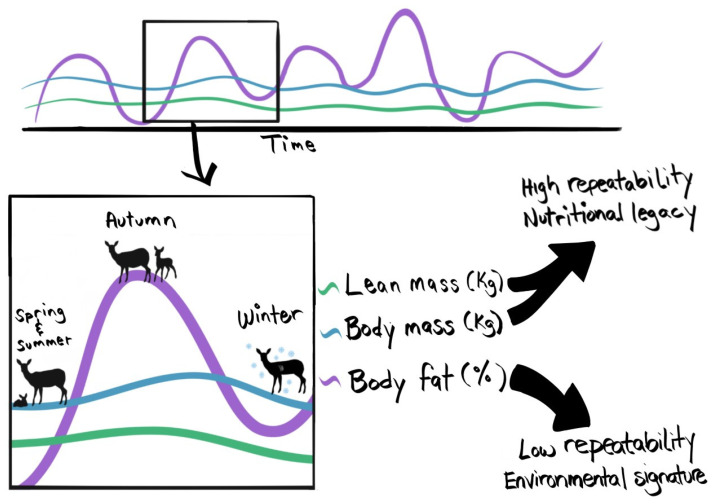
Conceptual representation of the dynamics of fat and lean mass in mule deer over time, where deer generally gained fat and protein reserves in the spring and summer and lost them over late fall and winter. Mule deer give birth in late spring and nurse their offspring throughout the summer. In the autumn, offspring are nutritionally independent. Our results suggest high repeatability of lean and body mass and low repeatability of body fat.

Repeatability may reveal the degree to which intrinsic (i.e., heritable) or extrinsic (i.e., environmental) factors drive phenotypic plasticity in a trait [55]. The repeatability of nutritional biomarkers should reflect how much that trait corresponds with environmental variation versus more intrinsic characteristics. We evaluated the degree of repeatability of three nutritional biomarkers (i.e., lean mass, body mass, and body fat) across eight years during spring and autumn in mule deer. In support of our hypothesis associated with the lability of various energy stores (H1), lean mass was most repeatable, followed by body mass, and body fat was least repeatable (P1; Figure 1). Consistent with our hypothesis of seasonal fluctuations in repeatability (H2), repeatability in biomarkers differed between seasons. In contrast to our prediction (P2), repeatability was greater in the autumn than in spring for all biomarkers, indicating that variability in environmental conditions in winter may have a larger influence on spring conditions, compared with the influence of conditions in summer on autumn conditions. As predicted, lean mass and body mass were more repeatable in autumn than in spring (P3). Notably, repeatability in nutritional biomarkers differed markedly, with lean mass and body mass being highly repeatable (>0.50; Figure 1) and body fat having low repeatability (<0.30; Figure 1). High repeatability suggests that lean mass and body mass are heritable traits that lack influence from short-term environmental stochasticity, whereas low repeatability of body fat supports that it is almost exclusively a product of the animal’s experience from the recent past (Figure 3). 

Variation in environmental conditions may have driven lower repeatability during spring compared with autumn across all biomarkers in our system. To survive harsh winters, mule deer, like other temperate ruminants, are metabolically preprogrammed to undergo a negative energy budget through a decrease in metabolism and loss of body reserves [56]. Importantly, these metabolic adaptations operate in a state-dependent manner in line with the risk-sensitive allocation of fat reserves [57]. Beyond animal state, variation in the catabolism of somatic reserves to meet energetic demands is a function of forage conditions and snow depth on the winter range [58,59]. Winter severity varied considerably during our study; severe winter conditions are more energetically costly because of locomotion through snow, thermoregulatory stress, and limitations to forage access and availability [59,60,61]. With less flexibility in resource allocation during winter (i.e., allocation focused primarily on survival and less on reproduction), combined with the stochasticity in winter severity, nutritional biomarkers become less repeatable as they become more reflective of extrinsic factors. Variation in winter conditions may influence the amount of body fat animals need to catabolize in a given winter to meet the energetic costs of survival, which we suspect contributed to the low repeatability in spring fat. While the potential for greater between-animal (i.e., population-level) variance of body fat was evident in autumn (Table 2, Figure 2), it may be that spring body fat levels are simply driven by environmental conditions (e.g., winter range conditions and severity) and have low repeatability. Alternatively, given that the population variance was so low, spring fat levels may indicate a population-level threshold, or set point, that, in the absence of severe winters, animals are metabolically programmed to reach in late winter [62]. Considering a metabolically programmed set point, repeatability may still be quite low but within a relatively small range, an assertion that is supported by the high coefficient of variation in spring fat. 

Set points can be influenced, in part, by environmental factors that determine the somatic reserves necessary to meet long-term metabolic requirements within the context of the system within which an animal resides [16]. Nutritional indices may differ dramatically across populations—how animals catabolize and accrete resources to meet energetic and metabolic demands may indicate the synchronization of an animal’s physiology with its environment. For example, average fat levels in autumn were 10.8% and dropped to 4.5% in the spring for our study population during an 8-year study, which is markedly different from the average decline in body fat from 9.7% in autumn to 7.2% in spring for mule deer in the Sierra Nevada of California during a 7-year study [22]. Compared with mule deer in the Wyoming Range, mule deer occupy relatively dry and unproductive summer ranges, and winters are mild in the Sierra Nevada [61]. Considering summer range quality dictates risk-sensitive allocation to a higher degree than winter severity, the less dramatic fluctuations between seasons in body fat of mule deer in the Sierra Nevada may be indicative of their environments and the resources they need to meet metabolic demands across seasons. If the metabolic programming of animals becomes mismatched to their environment because of unexpected, stochastic changes in conditions, it may come at a significant fitness cost [63]. Therefore, spring fat also may be one of the most important biomarkers for measuring responses and degree of plasticity to environmental change. 

Our assessment herein asserts that body fat is the most responsive of the three biomarkers to recent environmental conditions. Nevertheless, other biomarkers may still provide insight into individual-level variation but, importantly, at a different scope of inference. Body mass, an appealing biomarker because of its ease of acquisition, represents a composite measure of multiple biomarkers, including lean mass and body fat, while also incorporating animal size and ingesta. When interpreted cautiously, body mass may be informative to an animal’s fitness. Fluctuations in body mass between seasons may be driven primarily by changes in body fat. Thus, seasonal changes in body mass may be a useful proxy of environmental drivers of nutrition when fat measurements are unavailable but will notably be less responsive to extrinsic factors. Further, body mass has been shown to influence the survival and reproduction of large herbivores [20,37] and, in some instances, may be more predictive of survival than body fat [22].

Size or performance of animals can be reflective of conditions during the year they were born (i.e., cohort effects) and are a population-level example of cross-generational effects of maternal nutrition [64,65,66] that can yield disparities in reproduction and survival advantages between large- and small-bodied animals [19,20,31,36]. Adult body mass is underpinned by maternal and grandmaternal effects [67], where the nutritional status of the mother during gestation can determine the lifetime trajectory of growth for an animal, regardless of nutritional experiences thereafter [68,69,70,71]. For example, offspring born to mothers in poor nutritional condition may be subject to a negative maternal effect and, thus, will express the consequence of their mother’s nutritional condition during gestation for their entire life [69]. As a result, animals born to mothers in poor condition may be stunted in body mass regardless of the body fat that they are accreting and catabolizing through different seasons. Therefore, body mass and lean mass may be more useful in reflecting a historical nutritional legacy and the maternal condition during the year an animal was born than as a biomarker of current environmental conditions. Knowing how nutritional legacy, genetics, and environment contribute to potential biomarkers is key to their appropriate interpretation and scope of inference, which should be strongly considered for the most readily accessible biomarkers, such as body mass.

## 5. Conclusions

The repeatability of biomarkers can elucidate the scale at which they operate and, thus, provide insight into the underlying environmental factors that drive them [9]. Lean mass and body mass were highly repeatable in both autumn (0.72 and 0.64, respectively) and spring (0.61 and 0.53, respectively), which indicates a lack of sensitivity to environmental conditions and is more a reflection of heritability (via classic inheritance and nutritional legacy). Body fat had low levels of repeatability in both autumn (0.22) and spring (0.01), which indicates a strong influence of the environment in the preceding season on the amount of body fat that an animal accrues. Despite relatively large differences in the degree of repeatability across nutritional biomarkers, metrics of nutrition are often used interchangeably to investigate questions of nutritional relationships in wild animals [29,30]. Our results highlight the importance of understanding the underlying mechanisms that influence nutritional biomarkers—we strongly encourage careful consideration of the environmental drivers of each biomarker when estimating the nutritional status of individuals.

## Figures and Tables

**Table 1:** Mean unscaled values and median scaled repeatability estimates with 95% credible intervals for lean mass (ingesta-free, fat free body mass; IFFFBMass), body mass, and body fat (ingesta-free body fat; % IFBFat) and in female mule deer in the Wyoming and Salt Ranges, Wyoming, USA, between 2013 and 2021.

Biomarker	Season	Mean	Repeatability		
			Estimate	2.5% CRI	97.5% CRI
**Lean mass (kg)**	**Autumn**	60.4	0.72	0.65	0.78
	**Spring**	55.0	0.61	0.53	0.68
**Body mass (kg)**	**Autumn**	74.2	0.64	0.56	0.71
	**Spring**	65.1	0.53	0.45	0.61
**Body fat (% IFBFat)**	**Autumn**	10.81	0.22	0.11	0.33
	**Spring**	4.48	0.01	0.00	0.07

**Table 2:** Variance estimates with 95% credible intervals for lean mass (ingesta-free, fat free body mass), body mass, and body fat (ingesta-free body fat; % IFBFat) in female mule deer in the Wyoming Range, Wyoming, USA, between 2013 and 2021.

Biomarker	Season	Between-Animal Variance (Population)			Within-Animal Variance (Individual)		
		Estimate	2.5% CRI	97.5% CRI	Estimate	2.5% CRI	97.5% CRI
**Lean mass (kg)**	**Autumn**	0.37	0.29	0.49	0.18	0.12	0.17
	**Spring**	0.28	0.22	0.37	0.15	0.16	0.21
**Body mass (kg)**	**Autumn**	0.35	0.26	0.46	0.20	0.17	0.23
	**Spring**	0.22	0.17	0.30	0.20	0.17	0.23
**Body fat (% IFBFat)**	**Autumn**	0.21	0.10	0.34	0.74	0.63	0.87
	**Spring**	<0.01	<0.01	0.02	0.24	0.21	0.27

## Data Availability

Data was not publicly available at the time of publication; data requests can be made to Kevin Monteith.

## Supplementary Materials

The following supporting information (R Code) can be downloaded at: https://github.com/tlasharr/RepeatabilityBayes, accessed on 20 January 2022.
